# Mixed *Plasmodium* Infections: Report of Four Cases from the Military Hospital of Tunis

**DOI:** 10.12688/f1000research.173540.1

**Published:** 2025-12-10

**Authors:** Latifa Mtibaa, Souha Hannachi, Zeinab Denden, Rym Abid, Riadh Battikh, Boutheina Jemli

**Affiliations:** 1 Laboratory of Parasitology, Hôpital Militaire Principal d'Instruction de Tunis, Tunis, Tunis, 1008, Tunisia; 2 University of Tunis El Manar Faculty of Medicine of Tunis, Tunis, Tunis, Tunisia; 3 Infectious disease, Hôpital Militaire Principal d'Instruction de Tunis, Tunis, Tunis, Tunisia; 4 University of Monastir Faculty of Pharmacy of Monastir, Monastir, Monastir, Tunisia

**Keywords:** Malaria, mixed infection, Plasmodium, military

## Abstract

**Background:**

Malaria is a life-threatening vector-borne disease caused by five species of the genus *Plasmodium (P.)* It can present as mixed infections that remain underreported and pose diagnostic, therapeutic and prognostic challenges.

The objective of the present study was to describe the epidemiological, clinical, and biological features of four cases of mixed *Plasmodium* infection.

**Methods:**

This retrospective descriptive study included cases of mixed *Plasmodium* infections diagnosed at the Parasitology Laboratory of the Military Hospital of Tunis between 2022 and 2025. Diagnosis was established using May–Grünwald–Giemsa–stained thin blood smears, Giemsa–stained thick smears, and rapid diagnostic tests. *Plasmodium* species were identified according to standard microscopic morphological criteria.

**Results:**

We report four cases of mixed *Plasmodium* infection diagnosed in military personnel returning from deployment in Central Africa. The patients were aged 31–52 years (mean age 38.8 years) with sex ratio 3. They presented within 10–30 days post-return with fever (38–42 °C) associated with headache, myalgia, abdominal pain, and/or vomiting. All had adhered to malaria chemoprophylaxis, and three reported no prior malaria episodes, while one patient had a history of six previous attacks. Rapid diagnostic tests were positive for pan-Plasmodium pLDH in all cases; two patients were also positive for HRP2. Peripheral blood smears confirmed mixed infections. Parasitemia was generally low (<1% in three cases, 1% in one case). Treatment consisted of artemether–lumefantrine (Coartem ^®^) for all patients, combined with primaquine for those with *P. ovale* infection. Clinical evolution was favorable in all cases, and follow-up blood smears on days 3, 7, and 28 confirmed parasitological clearance, with only rare degenerated trophozoites observed in one patient on day 3.

**Conclusion:**

Mixed *Plasmodium* infections can influence disease severity, treatment outcomes, and drug resistance. Accurate diagnosis and radical therapy are keys to preventing relapses.

## Introduction

Malaria is a life-threatening vector-borne disease caused by five species of the genus *Plasmodium (P.).* The most common species involved in human malaria are *Plasmodium falciparum*, *Plasmodium vivax, Plasmodium malariae, Plasmodium ovale*, and *Plasmodium knowlesi.* The infection involves usually a single species. Mixed *Plasmodium* infection (MPI) refers to the simultaneous infection with more than one species of the malaria-causing parasite *Plasmodium.* ^1^

MPI are undereported in the literature but relatively common, especially in regions where multiple *Plasmodium* species coexist. ^1, 2^ In some African and Asian regions, mixed infections are found in up to 4% of malaria-positive cases. Although, this number may be underestimated due to diagnostic challenges because traditional microscopy may miss one of the infecting species, especially when there is a dominance of one species in the blood. ^1, 3^

MPI can be associated with severe malaria. ^4^ Notably, severe complications in mixed infections may involve severe anemia, pulmonary failure, and renal impairment. Furthermore, patients with mixed infections may experience a higher likelihood of multiple organ failure compared to those with single-species infections. ^5^

After treatment, patients with MPI have a higher risk of malaria recurrence, especially within the first month following therapy, as compared to those with single-species infections. ^6^ The choice of antimalarial treatment is critical, to ensure clearance of all *Plasmodium* species and prevent relapses. ^5, 6^

Given the diagnostic, therapeutic, and prognostic difficulties, we report in this study the epidemiological, clinical, and biological characteristics of four cases of MPI diagnosed in military personnel returning from deployment and treated at the Military Hospital in Tunis.

## Methods

This retrospective descriptive study included cases of mixed *Plasmodium* infections diagnosed at the Parasitology Laboratory of the Military Hospital of Tunis between 2022 and 2025. Diagnosis was established using May–Grünwald–Giemsa–stained thin blood smears, Giemsa–stained thick smears, and rapid diagnostic tests. *Plasmodium* species were identified according to standard microscopic morphological criteria.

## Results

Over a four-year period (2022-2025), we identified four cases of MPI among a total of 175 cases of imported malaria in Tunisian military personnel returning from missions in endemic areas. This represents a prevalence of 2.2%. All four cases had stayed in Central Africa and were recorded in 2024 (n = 3) and 2025 (n = 1).

The patients were aged 31–52 years (mean age 38.8 years) with sex ratio 3. They presented within 10–30 days post-return with fever (38–42°C) associated with headache, myalgia, abdominal pain, and/or vomiting. All had adhered to malaria chemoprophylaxis, and three reported no prior malaria episodes, while one patient had a history of six previous attacks (patient 3).

Rapid diagnostic tests (RDT) were positive for pan- *Plasmodium* pLDH (PAN) in all cases; two patients were also positive for HRP2 (specific for *P. falciparum*). Peripheral blood smears confirmed mixed infections: *P. falciparum and P. malariae* in one patient, *P. ovale* and *P. malariae* in one patient, and *P. falciparum* and *P. ovale* in two patients. Parasitemia was generally low (<1% in three cases, 1% in one case).

Treatment consisted of artemether–lumefantrine (Coartem ^®^ 20 mg/120 mg) for all patients, administered at a dosage of 4 tablets at H0, H8, H24, H36, H48, and H60. It was combined with primaquine in three patients (those with P. ovale infection), at a dosage of 30 mg/day for 15 days. All patients adhered fully to the prescribed treatment, and no adverse or unanticipated events were observed during therapy.

Clinical evolution was favorable in all cases, and follow-up blood smears on days 3, 7, and 28 confirmed parasitological clearance, with only rare degenerated trophozoites observed in one patient on day 3.

**Figure 1.**
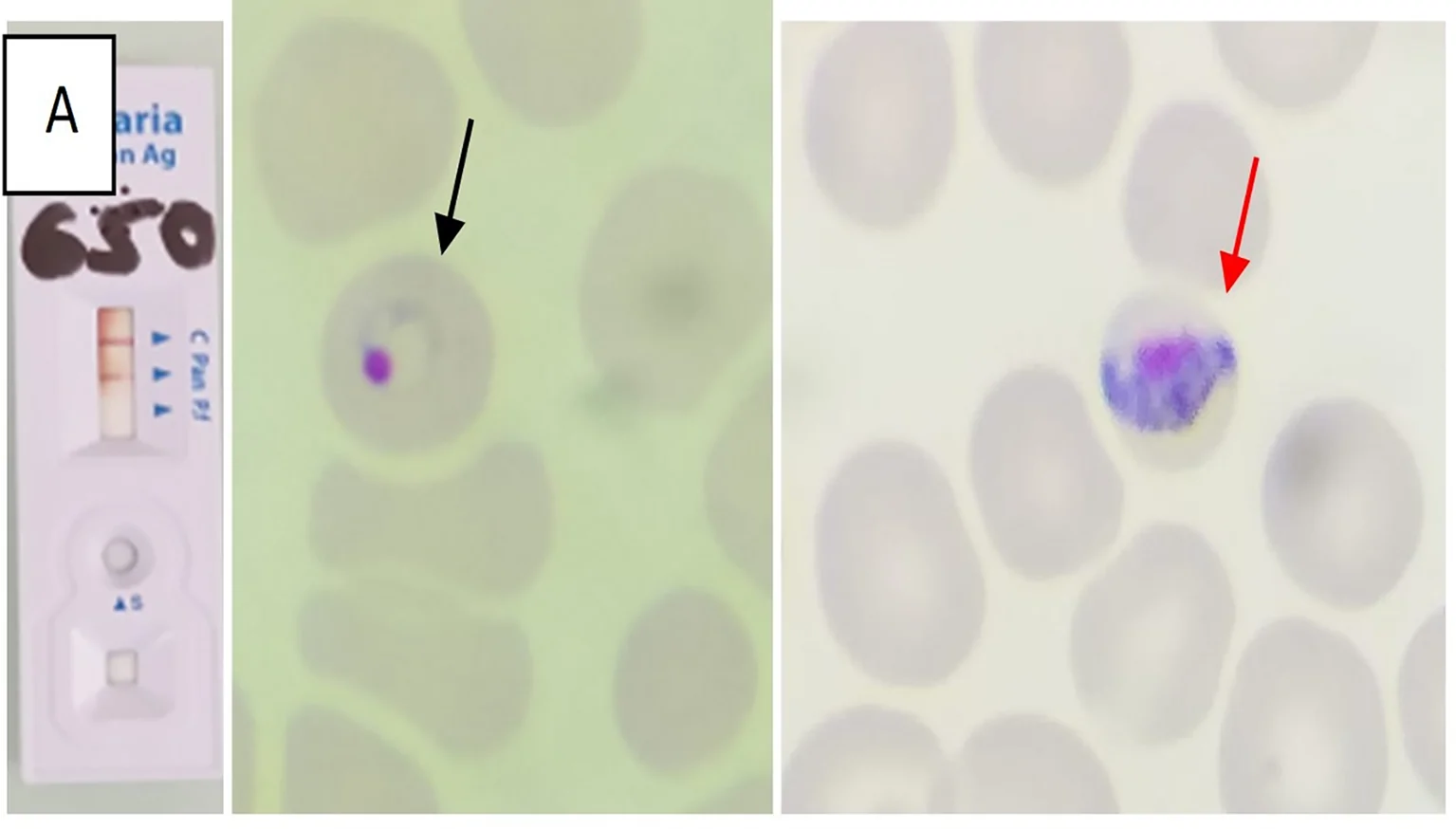
A: RDT result: PAN +; Black arrow: trophozoite of *Plasmodium falciparum*; Red arrow: Trophozoite of *Plasmodium malariae* (case 1).

**Figure 2.**
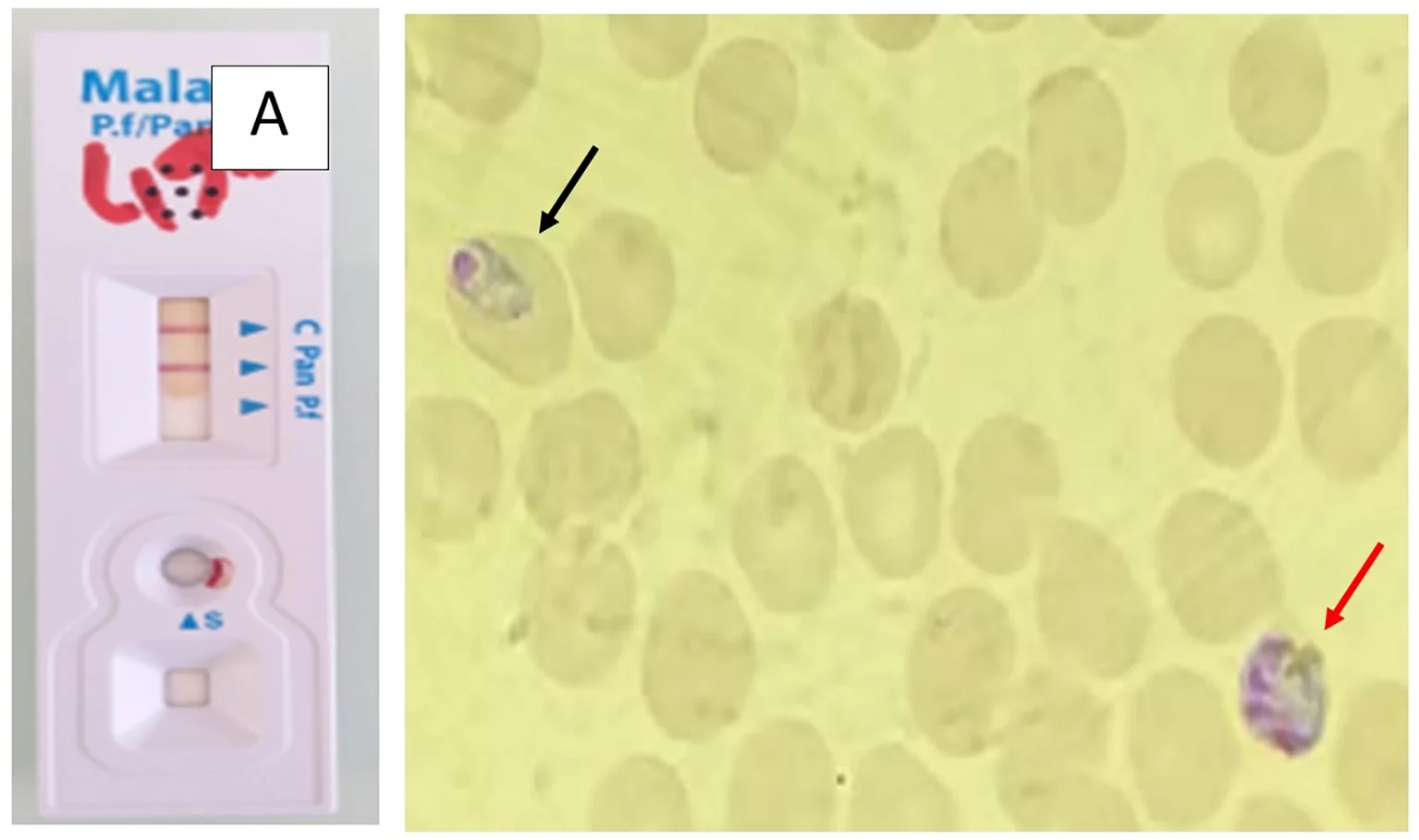
A: RDT result: PAN +; Black arrow: trophozoite of *Plasmodium ovale;* Red arrow: Schizont of *Plasmodium malariae* (case 2).

**Figure 3.**
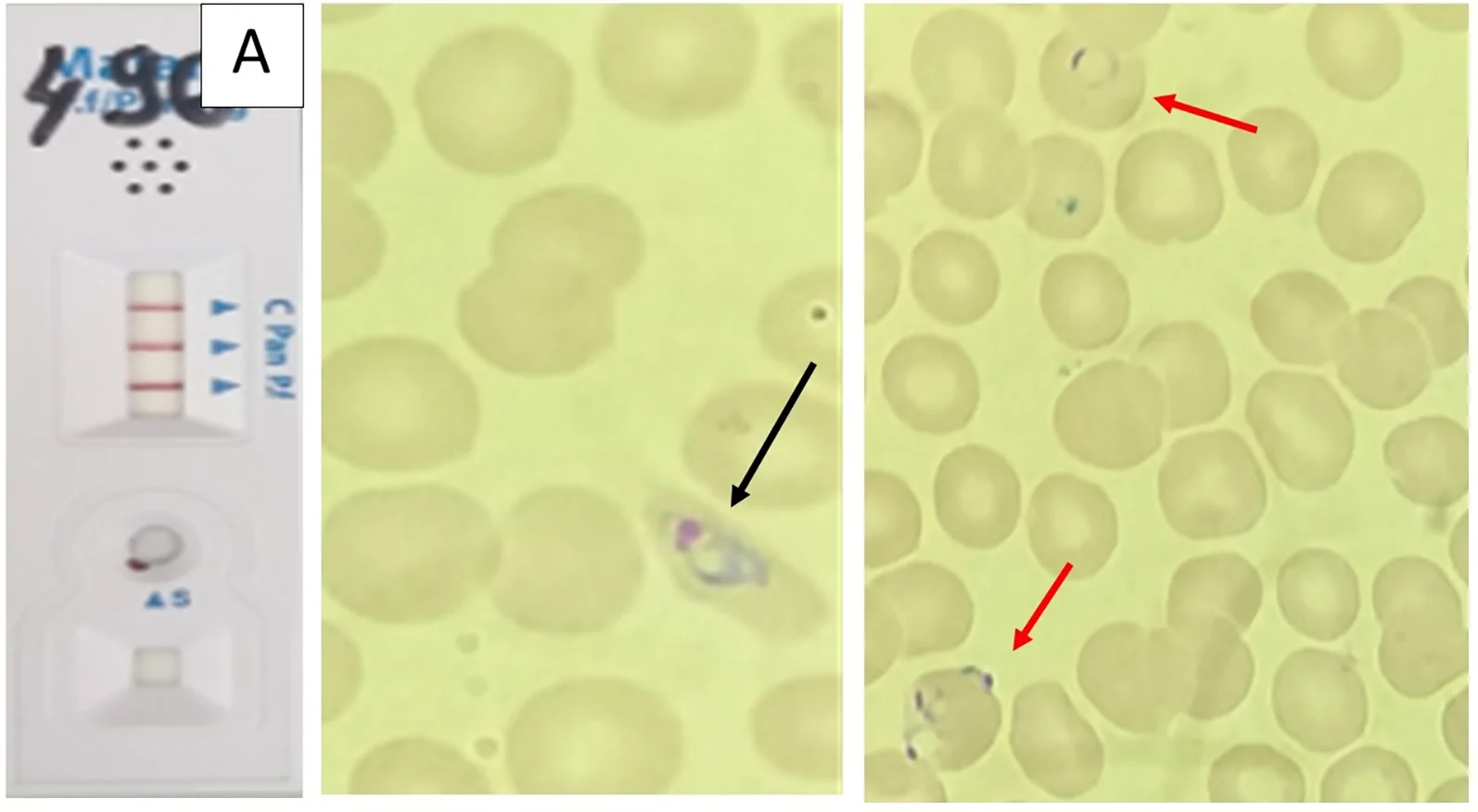
A: RDT result: PAN + Pf; Black arrow: Trophozoite *Plasmodium ovale*; Red arrows: trophozoite of *Plasmodium falciparum* (case 3).

**Figure 4.**
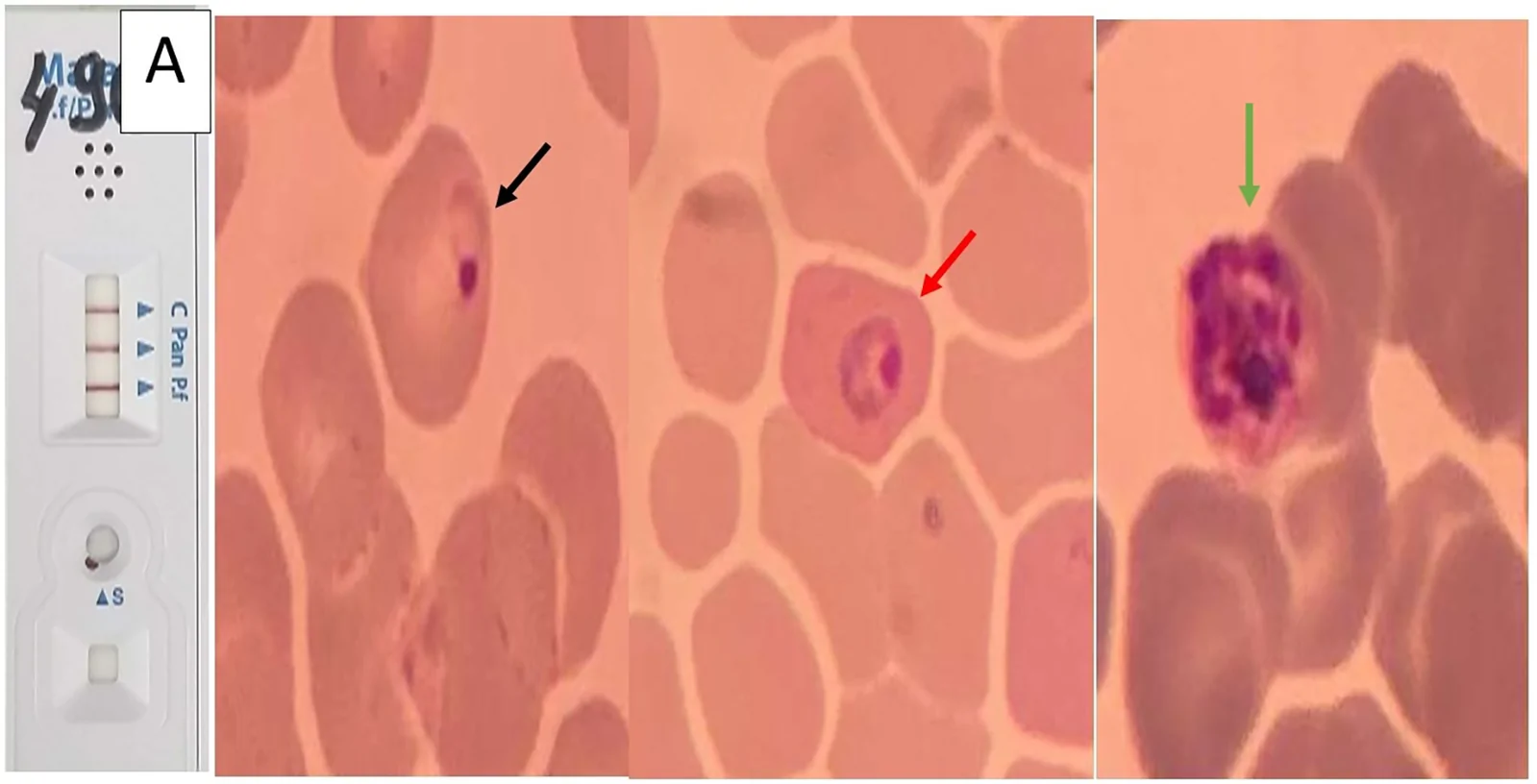
A: RDT result: PAN + Pf; Black arow: trophozoite de *P. falciparum*; Red arrow: trophozoite de *P. ovale*; Green arrow: schizont de *P. ovale* (case 4).

Tables 1 and 2 summarized the main epidemiological, clinical, and biological features of the four patients. Figures 1, 2, 3, and 4 correspond to the parasitological diagnostic results of patients 1 to 4, respectively, including rapid diagnostic tests and blood smears.

**Table 1.** Epidemiological, clinical, and parasitological features of the four patients with mixed *Plasmodium* infections.

Patient	1	2	3	4
Date of diagnosis	25/01/2024	18/02/2024	20/02/2024	26/05/2025
Age/sex	33/M	52/M	31/M	39/F
Return from Central Africa (days)	10	30	30	15
Clinical presentation	Fever 41°C, abdominal pain, headache, myalgia	Fever 42°C, chills, asthenia	Fever 40°C, headache, vomiting; 6 prior malaria episodes	Fever 38°C, headache, myalgia
RDT result	PAN (+)	PAN (+)	HRP2 (+), PAN (+)	HRP2 (+), PAN (+)
Blood smear findings	*P. falciparum + P. malariae* (trophozoites)	*P. ovale + P. malariae* (trophozoites, schizonts, gametocytes)	*P. falciparum* (trophozoites) + *P. ovale* (trophozoites, schizonts, gametocytes)	*P. falciparum* (trophozoites) + *P. ovale* (trophozoites, schizonts)
Parasitemia	1%	<1%	<1%	<1%
Treatment	Coartem ^®^	Coartem ^®^ + Primaquine	Coartem ^®^ + Primaquine	Coartem ^®^ + Primaquine
Outcome (D3-D7-D28 smears)	Negative	Negative	Negative	Rare degenerated trophozoites at D3 Negative at D7–D28

**Table 2.** Biological characteristics of the four patients with mixed *Plasmodium* infections.

Complete Blood count (CBC), differential white blood cells (WBCs) count and coagulation tests					
	Case 1	Case 2	Case 3	Case 4	Reference interval
Blood group	-	AB positif	O positif	AB negatif	-
Hemoglobin	14.6	11,7	15,2	14	12-16 g/dl
Hematocrit	42.9%	35.2		42	37-47%
Red blood cells	4.8	4.18		5.14	4.2-5.5 X 10 ^12^/L
MCV	89.3	84.2	83.3	81.7	80-94 fl
MCH	34.1	27.9	28	27.2	27-32 pg
White blood cell count X 10 ^9^/L	8.0	4.8	10.5	9.3	4-11 X 10 ^9^/L
Neutrophils X 10 ^9^/L	6.6	3.9	9.0	8.1	1.5-8 X 10 ^9^/L (40-75%)
Lymphocytes X 10 ^9^/L	0.9	0.4	1	0.6	1-4 X 10 ^9^/L (20-45%)
Monocytes X 10 ^9^/L	0.5	0.5	0.5	0.5	0.1-0.7 X 10 ^9^/L (3-9%)
Eosinocytes X 10 ^9^/L	00	00	00	00	0.05-0.5 X 10 ^9^/L (0-6%)
Basophils X 10 ^9^/L	00	00	00	0.1	0.025-0.1 X 10 ^9^/L (0-1%)
Platelets X 10 ^9^/L	86 X 10 ^9^/L	37 X 10 ^9^/L	87 X 10 ^9^/L	48 X 10 ^9^/L	140-450 X 10 ^9^/L
Coagulation tests					
Prothrombin time	-	61%		90%	70-100%
International Normalized ratio	-	1.37		1.05	2-3
Biochemistry, liver function tests and inflammatory blood markers tests					
C- reactive protein	144	142	109	123	<6 mg/L
Erythrocyte sedimentation rate					0-20 mm/hour
Total Protein	7.2	6.3	7.7	6.9	6.4-8.2 g/dL
Albumin					3.4-5.0 g/dL
Na/K	134/3.6	131/3.6	131/4.1	136/3.9	136-145 mEq/L/3.5-5.1 mEq/L
Urea	7.5	5.3	5.2	6.4	7-18 mg/dL
Creatinin	89	97	69	65	60-105 μmol/L
AST (Aspartate Aminotransferase)	17	136	19	45	15-37 U/L
ALT (Alanine Aminotransferase)	17	118	20	45	14-36 U/L
Alkaline phosphatase	65	208	96	58	46-116 U/L
Gamma-glutamyl transferase	34	516	42	38	5-55 U/L
Total bilirubin	29	37	21	10	<20 μmol/L
Direct bilirubin	3	19	-	-	<5,1 μmol/L

## Discussion

This study provides a detailed description of four MPI in military personnel returning from Central Africa, with thorough documentation of clinical features, diagnostic tests, and follow-up parasitological clearance, which represents strength. Limitations include the small sample size, retrospective design, lack of molecular confirmation, and the exclusive focus on military personnel. Despite these constraints, the cases highlight that mixed infections can occur even under chemoprophylaxis, may present with low parasitemia, and require careful species identification.

MPI are underreported, but they are more common than previously thought, especially in regions where multiple species coexist. These infections may go undetected due to the dominance of one species or low parasitemia.

Studies in African and Asian endemic areas report prevalence rates ranging from 2% to 30%, depending on local transmission intensity and the sensitivity of diagnostic tools. ^7– 9^ The prevalence of MPI in our study was 2.2%. It reports four cases: one involving *P. falciparum* and *P. malariae*, one involving *P. ovale* and *P. malariae*, and two involving *P. falciparum and P. ovale.*

In Tunisia, MPI remain poorly documented. Bouratbine A. et al. reported five cases of MPI among 240 *Plasmodium* infections (1980-1995), including *P. falciparum–P. ovale* (2 cases), *P. falciparum–P. malariae* (2 cases), and *P. falciparum–P. vivax* (1 case). ^10^ Belhadj et al. found six cases of MPI among 291 *Plasmodium* infections (1991-2006), with a similar distribution: *P. falciparum–P. ovale* (4 cases), *P. falciparum–P. malariae* (1 case), and *P. falciparum–P. vivax* (1 case). ^11^ More recently, Siala et al. reported two cases of PMI: *P. falciparum–P. ovale* and *P. falciparum–P. malariae.* ^12^

MPI can result from several factors. Multiple *Anopheles* species may transmit different *Plasmodium* species simultaneously in a single inoculation, or a single *Plasmodium* species may be introduced through successive bites by infected mosquitoes. *P. vivax* and *P. ovale* can persist as liver hypnozoites, causing relapses, while *P. falciparum* may recrudesce due to drug-resistant forms. Incomplete treatment of a prior infection can also lead to persistent or mixed parasitemia. These mechanisms highlight the complexity of diagnosing and managing mixed malaria infections in endemic areas. ^4– 8^

Accurate identification of mixed infections is crucial for appropriate management. Peripheral blood smear microscopy remains the gold standard for species identification and quantification, allowing detection of multiple species. However, it may fail to detect low-density parasitemia or minor species. Rapid diagnostic tests (RDTs) are valuable for rapid screening, detecting pan-Plasmodium antigens and species-specific markers, but they have known limitations, as some minor infections may yield false-negative results. ^13, 14^ Molecular techniques, such as PCR especially multiplex, nested approaches or real-time PCR, offer superior sensitivity and specificity, capable of identifying submicroscopic infections and clarifying complex species combinations. ^1, 2, 15, 16^ Metagenomic next-generation sequencing (mNGS) is a powerful tool for detecting mixed infections, useful in atypical or severe cases where routine tests fail, but its cost and complexity limit malaria use in endemic regions. ^17^ Incorporating multiple diagnostic approaches can improve detection and guide targeted therapy.

Treatment of mixed infections should aim to eliminate all infecting species and prevent relapses. In our cases, artemether–lumefantrine was used for all patients, complemented by primaquine for *P. ovale* infections to target dormant liver stages. This combined approach resulted in rapid clinical recovery and confirmed parasitological clearance by day 28.

Appropriate management of mixed *Plasmodium* infection requires selecting antimalarial regimens effective against all infecting species. Artemisinin-based combination therapies (ACT) are effective against *P. falciparum* and non- *falciparum* species. Additional drugs such as primaquine are needed to eliminate hepatic stages of *P. vivax* and *P. ovale.* ^18^ Treatment should be based on an ACT with the addition of primaquine when indicated and once G6PD deficiency has been excluded for radical cure in cases involving *P. vivax* or *P. ovale.*

Early intervention and adherence to recommended treatment protocols are critical, as untreated or inadequately treated mixed infections may contribute to severe disease manifestations, prolonged illness, and potential development of antimalarial resistance. ^18, 19^ Our findings reinforce the need for clinicians to consider species-specific therapies in co-endemic settings.

Preventive strategies remain essential to reduce the incidence and impact of malaria, particularly for travelers and military personnel deployed in endemic areas. Effective measures include adherence to chemoprophylaxis, use of insecticide-treated bed nets, application of topical repellents, and prompt medical evaluation at the onset of fever or other malaria symptoms. Education and awareness of the risk of these infections are also vital. ^20^ Strengthening preventive programs and surveillance can help mitigate the burden of imported and co-endemic malaria cases.

## Conclusion

Mixed *Plasmodium* infections, frequently underdiagnosed, pose diagnostic, therapeutic, and prognostic challenges, particularly in travelers and military personnel returning from endemic areas. Our case series highlights the importance of combining microscopy, rapid diagnostic tests, and, when available, molecular methods to ensure accurate species identification. Timely administration of appropriate ACT complemented by primaquine for relapsing species, resulted in full recovery and parasitological clearance in all cases. Optimizing diagnostic approaches, adherence to treatment guidelines, and reinforcing preventive measures remain essential to reduce the burden and complications of mixed malaria infections.

## Consent

Written informed consent for the publication of clinical details was obtained from all patients.

## Data Availability

All data underlying the results are included within the article, and the CARE Checklist is openly available in public repositories (Mtibaa L. CARE checklist – Mixed Plasmodium infection [Data set]. Zenodo; 2025. DOI: https://doi.org/10.5281/zenodo.17650886 under a CC0 1.0 license.)
