# Mixed *Plasmodium* Infections: Report of Four Cases from the Military Hospital of Tunis

**DOI:** 10.12688/f1000research.173540.3

**Published:** 2026-09-08

**Authors:** Latifa Mtibaa, Souha Hannachi, Zeinab Denden, Rym Abid, Riadh Battikh, Boutheina Jemli

**Affiliations:** 1 Laboratory of Parasitology, Hôpital Militaire Principal d'Instruction de Tunis, Tunis, Tunis, 1008, Tunisia; 2 University of Tunis El Manar Faculty of Medicine of Tunis, Tunis, Tunis, Tunisia; 3 Infectious disease, Hôpital Militaire Principal d'Instruction de Tunis, Tunis, Tunis, Tunisia; 4 University of Monastir Faculty of Pharmacy of Monastir, Monastir, Monastir, Tunisia

**Keywords:** Malaria, mixed infection, Plasmodium, military

## Abstract

**Background:**

Malaria is a life-threatening vector-borne disease caused by five species of the genus *Plasmodium (P.)* It can present as mixed infections that remain underreported and pose diagnostic, therapeutic and prognostic challenges.

The objective of the present study was to describe the epidemiological, clinical, and biological features of four cases of mixed *Plasmodium* infection.

**Methods:**

This retrospective descriptive study included cases of mixed *Plasmodium* infections diagnosed at the Parasitology Laboratory of the Military Hospital of Tunis between 2022 and 2025. Diagnosis was established using May–Grünwald–Giemsa–stained thin blood smears, Giemsa–stained thick smears, and rapid diagnostic tests. *Plasmodium* species were identified according to standard microscopic morphological criteria.

**Results:**

We report four cases of mixed *Plasmodium* infection diagnosed in military personnel returning from deployment in Central African Republic. The patients were aged 31–52 years (mean age 38.8 years) with sex ratio 3. They presented within 10–30 days post-return with fever (38–42°C) associated with headache, myalgia, abdominal pain, and/or vomiting. All had adhered to malaria chemoprophylaxis, and three reported no prior malaria episodes, while one patient had a history of six previous attacks. Rapid diagnostic tests were positive for pan-Plasmodium pLDH in all cases; two patients were also positive for HRP2. Peripheral blood smears confirmed mixed infections. Parasitemia was generally low (<1% in three cases, 1% in one case). Treatment consisted of artemether–lumefantrine (Coartem ^®^) for all patients, combined with primaquine for those with *P. ovale* infection. Clinical evolution was favorable in all cases, and follow-up blood smears on days 3, 7, and 28 confirmed parasitological clearance, with only rare degenerated trophozoites observed in one patient on day 3.

**Conclusion:**

Mixed *Plasmodium* infections can influence disease severity, treatment outcomes, and drug resistance. Accurate diagnosis and radical therapy are keys to preventing relapses.

## Introduction

Malaria is a life-threatening vector-borne disease caused by five species of the genus *Plasmodium* (P.): *P. falciparum*, *P. vivax*, *P. malariae*, *P. ovale*, and *P. knowlesi.* While infection typically involves a single species, mixed *Plasmodium* infection (MPI) refers to the simultaneous presence of more than one species. ^1^

MPI are underreported but relatively common in areas where several *Plasmodium* species coexist. ^1^ ^,^ ^2^ In some African and Asian regions, mixed infections may represent up to 4% of malaria-positive cases, although this proportion is likely underestimated due to diagnostic limitations, particularly the reduced sensitivity of microscopy for detecting minor species in the presence of a dominant parasite. ^1^ ^,^ ^3^ Clinically, MPI have been associated with severe malaria manifestations, including severe anemia, respiratory distress, and renal impairment, and may increase the risk of multi-organ failure compared with single-species infections. ^4^ ^,^ ^5^ In addition, mixed infections have been linked to a higher risk of early recurrence after treatment. ^6^

Although malaria was officially eradicated in Tunisia in 1979, imported cases continue to be reported due to travel to endemic regions and the persistence of *Anopheles* vectors. Most imported cases originate from sub-Saharan Africa, particularly Côte d’Ivoire and the Democratic Republic of the Congo. ^7^ ^,^ ^8^ Among Tunisian military personnel, few studies have specifically addressed MPI, and epidemiological data remain limited. ^9^ A clear understanding of species distribution and epidemiological patterns is essential for accurate diagnosis, optimal treatment, and relapse prevention.

Given the diagnostic and therapeutic challenges posed by MPI, we report the epidemiological, clinical, and biological characteristics of four cases diagnosed in Tunisian military personnel returning from deployment and managed at the Military Hospital in Tunis.

## Methods

This retrospective descriptive study included cases of mixed *Plasmodium* infections diagnosed at the Parasitology Laboratory of the Military Hospital of Tunis between 2022 and 2025. The denominator for prevalence calculation included all imported malaria cases diagnosed during the study period.

Diagnosis was established using May–Grünwald–Giemsa–stained thin blood smears and Giemsa-stained thick smears, examined by experienced microscopists. Species identification was based on standard morphological criteria, including parasite size and shape, presence of Schüffner’s stippling, band forms, schizont morphology, and gametocyte characteristics. Parasitaemia was estimated on the thin blood smear and expressed as the percentage of parasitized red blood cells. It was calculated using the following formula:

$$\text{Parasitaemia (\%)}=\frac{\text{number of parasitized red blood cells (RBCs)}}{\left(\text{number of RBCs per field × number of fields examined}\right)}\times 100.$$

An average of approximately 250 red blood cells per microscopic field was considered for this estimation.

Rapid diagnostic tests were performed using the STANDARD™ Q Malaria P.f/Pan Ag (SD Biosensor ^®^), which detects histidine-rich protein 2 (HRP2) specific for *Plasmodium falciparum* and pan- *Plasmodium* lactate dehydrogenase (pLDH). Tests were conducted according to the manufacturer’s instructions.

All four patients received malaria chemoprophylaxis with doxycycline (100 mg once daily), initiated 1–2 days before travel to endemic areas, continued throughout deployment in Central African Republic, and maintained for four weeks after return, in accordance with current WHO and Tunisian military health recommendations. Information regarding malaria chemoprophylaxis, including the regimen, timing, duration, and adherence, was obtained through structured medical interviews upon return; compliance was self-reported and could not be objectively verified.

In addition to chemoprophylaxis, preventive measures included the use of insecticide-treated bed nets, topical mosquito repellents (DEET-based), and protective clothing.

All patients received artemether–lumefantrine (Coartem ^®^ 20 mg/120 mg) in accordance with the WHO 2025 malaria treatment guidelines. Those with *P. ovale* infection received an additional 15-day course of primaquine for radical cure, as recommended by the WHO. Patients on primaquine were monitored using serial complete blood counts (CBC) to detect any signs of hemolysis; no quantitative G6PD testing was performed.

**Ethics statement:** This retrospective study used anonymised patient data. According to institutional regulations, formal ethical approval was not required. The study was conducted in accordance with the principles of the Declaration of Helsinki.

## Results

Over a four-year period (2022-2025), we identified four cases of MPI among a total of 175 cases of imported malaria in Tunisian military personnel returning from missions in endemic areas. This represents a prevalence of 2.3%. Among the remaining 171 imported malaria cases, species distribution was as follows: *Plasmodium falciparum* (40%), *Plasmodium ovale* (57%), and *Plasmodium malariae* (3%).

All four cases had stayed in **Central African Republic** and were recorded in 2024 (n = 3) and 2025 (n = 1). Patients were aged 31–52 years (mean age 38.8 years) with a sex ratio of 3 M: 1F.

They presented within 10–30 days post-return with fever (38–42°C) associated with headache, myalgia, abdominal pain, and/or vomiting. All had adhered to malaria chemoprophylaxis, and three reported no prior malaria episodes, while one patient had a history of six previous attacks (patient 3). Previous episodes were treated with artemisinin-based combination therapy without primaquine in earlier episodes, which may explain relapses related to dormant liver hypnozoites.

Rapid diagnostic tests (RDT) were positive for pan- *Plasmodium* pLDH (PAN) in all cases; two patients were also positive for HRP2 (specific for *P. falciparum*). Peripheral blood smears confirmed mixed infections: *P. falciparum and P. malariae* in one patient, *P. ovale* and *P. malariae* in one patient, and *P. falciparum* and *P. ovale* in two patients. In our series, *Plasmodium falciparum* was morphologically dominant in cases 1, 3, and 4, whereas *Plasmodium ovale* predominated in case 2.

Parasitemia was generally low (<1% in three cases, 1% in one case). Low parasitaemia despite symptoms may be explained by partial immunity related to repeated exposure in endemic areas, prior malaria episodes (notably in patient 3), and chemoprophylaxis use, which may attenuate parasite multiplication without preventing infection.

Treatment consisted of Coartem ^®^ 20 mg/120 mg for all patients, administered as 4 tablets at H0, H8, H24, H36, H48, and H60. Primaquine was added for three patients with *P. ovale* infection, at a dosage of 30 mg/day for 15 days. All patients adhered fully to the prescribed treatment. Patients receiving primaquine were monitored by serial complete blood counts to detect any signs of hemolysis. No hemolysis or significant changes in hemoglobin levels were observed, and no adverse or unexpected events occurred during therapy.

Clinical evolution was favorable in all cases, and follow-up blood smears on days 3, 7, and 28 confirmed parasitological clearance, with only rare degenerated trophozoites observed in one patient on day 3.

**Figure 1.**
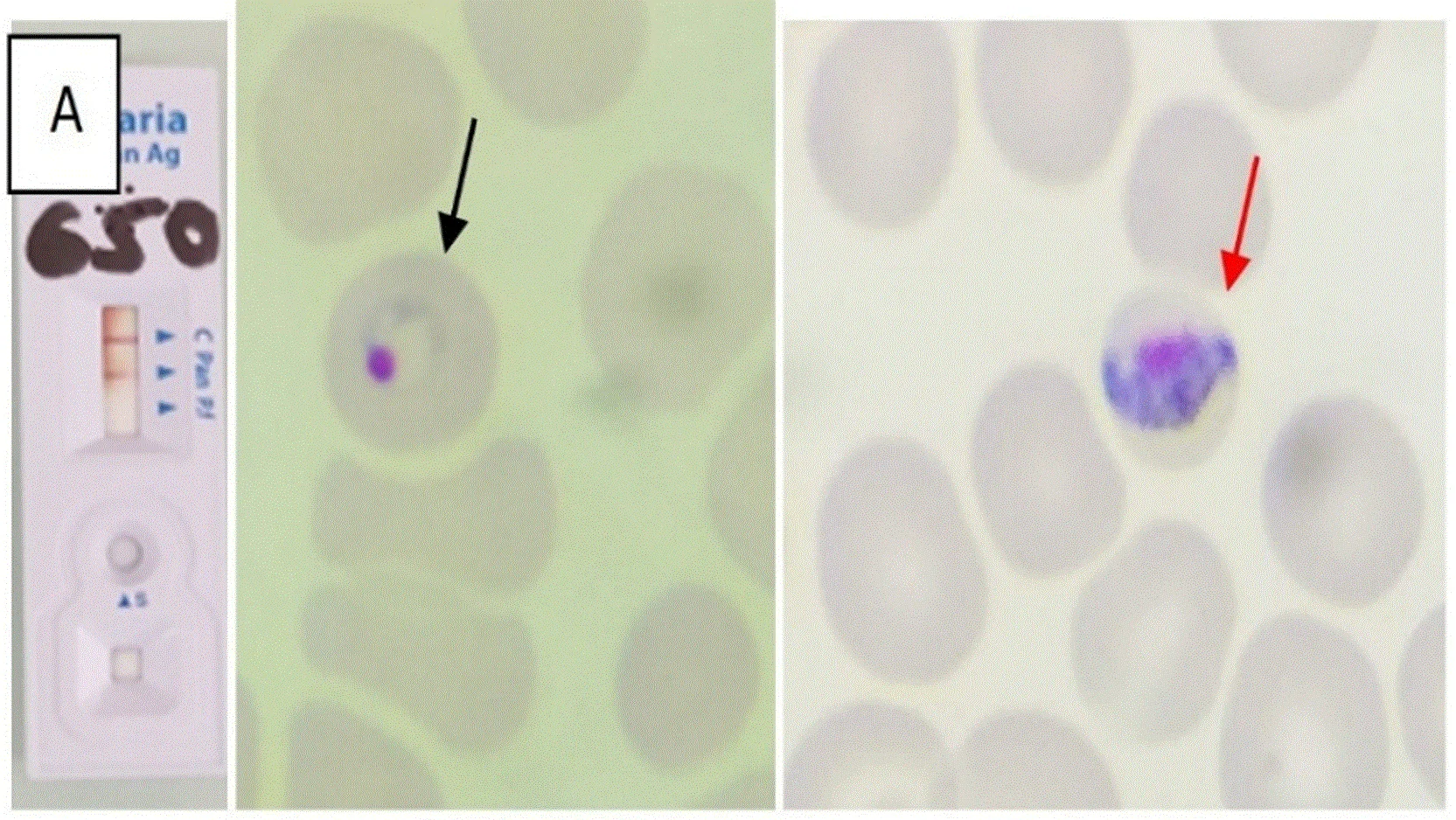
RDT and microscopic findings in case 1. A: PAN positive RDT; *P. falciparum *trophozoite (black arrow) and *P. malariae* trophozoite (red arrow) on thin blood smear.

**Figure 2.**
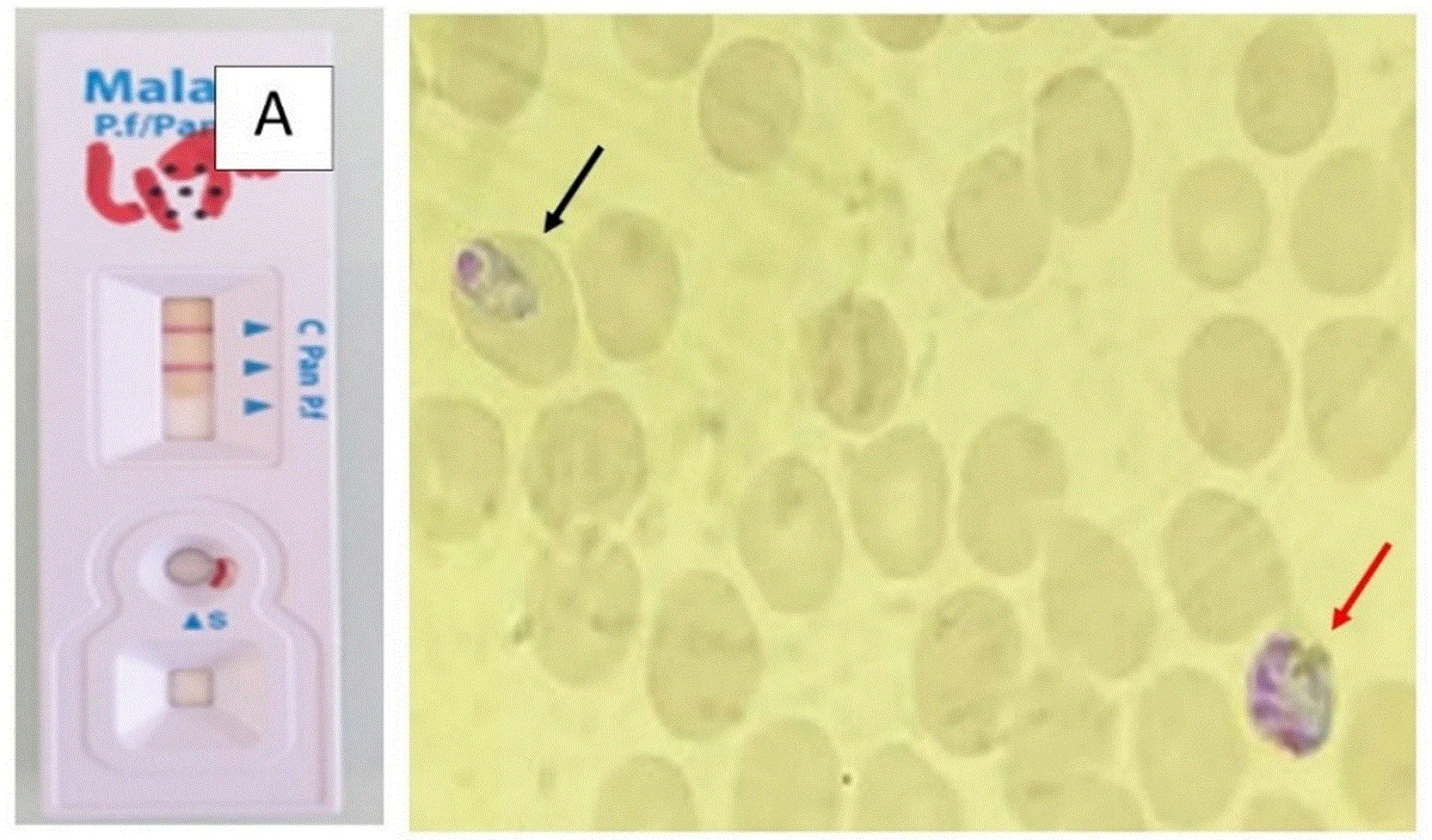
RDT and microscopic findings in case 2. A: PAN positive RDT; *P. ovale* trophozoite (black arrow) and *P. malariae* schizont (red arrow) on thin blood smear.

**Figure 3.**
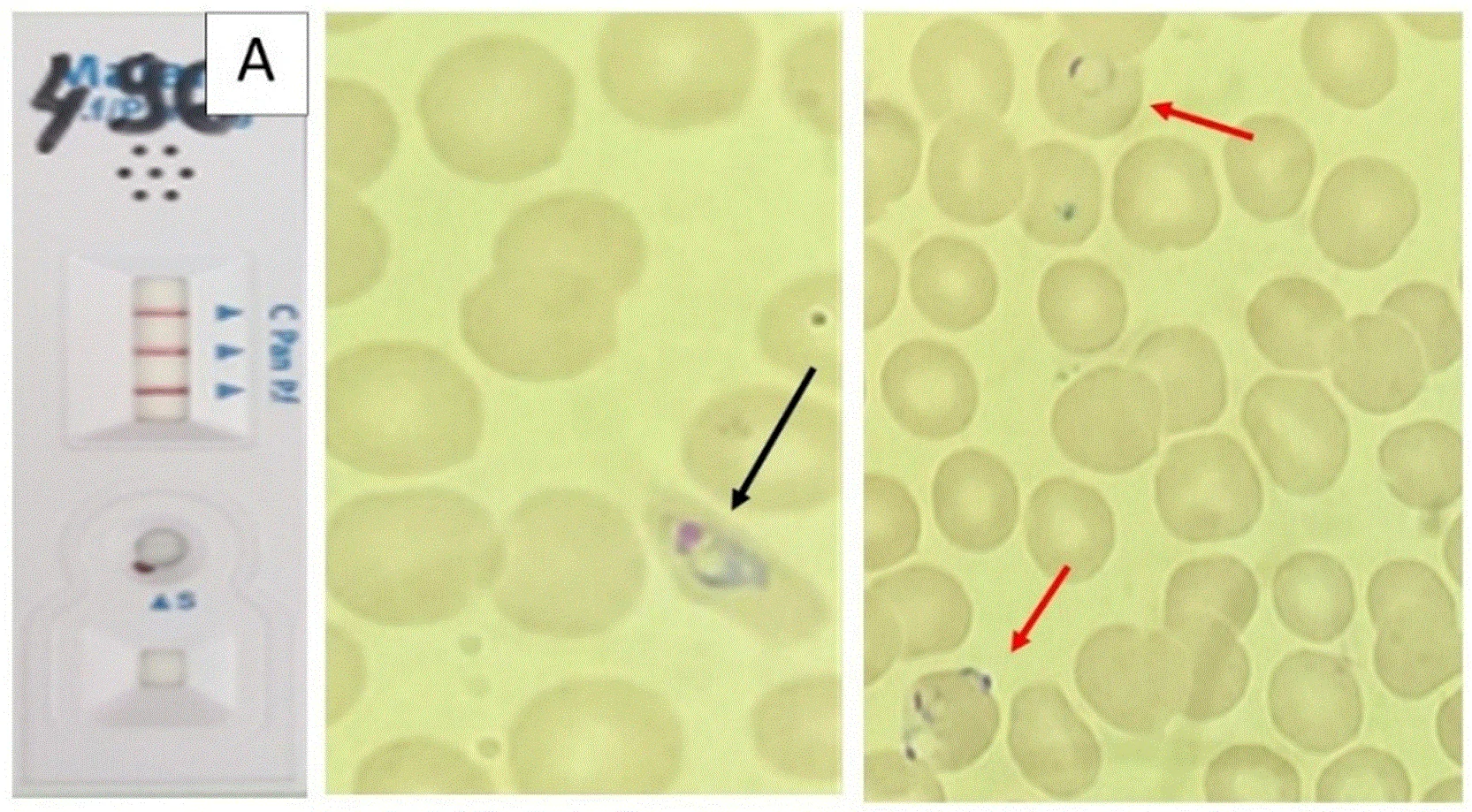
RDT and microscopic findings in case 3. A: PAN/Pf positive RDT; *P. ovale* trophozoite (black arrow) and *P. falciparum* trophozoites (red arrows) on thin blood smear.

**Figure 4.**
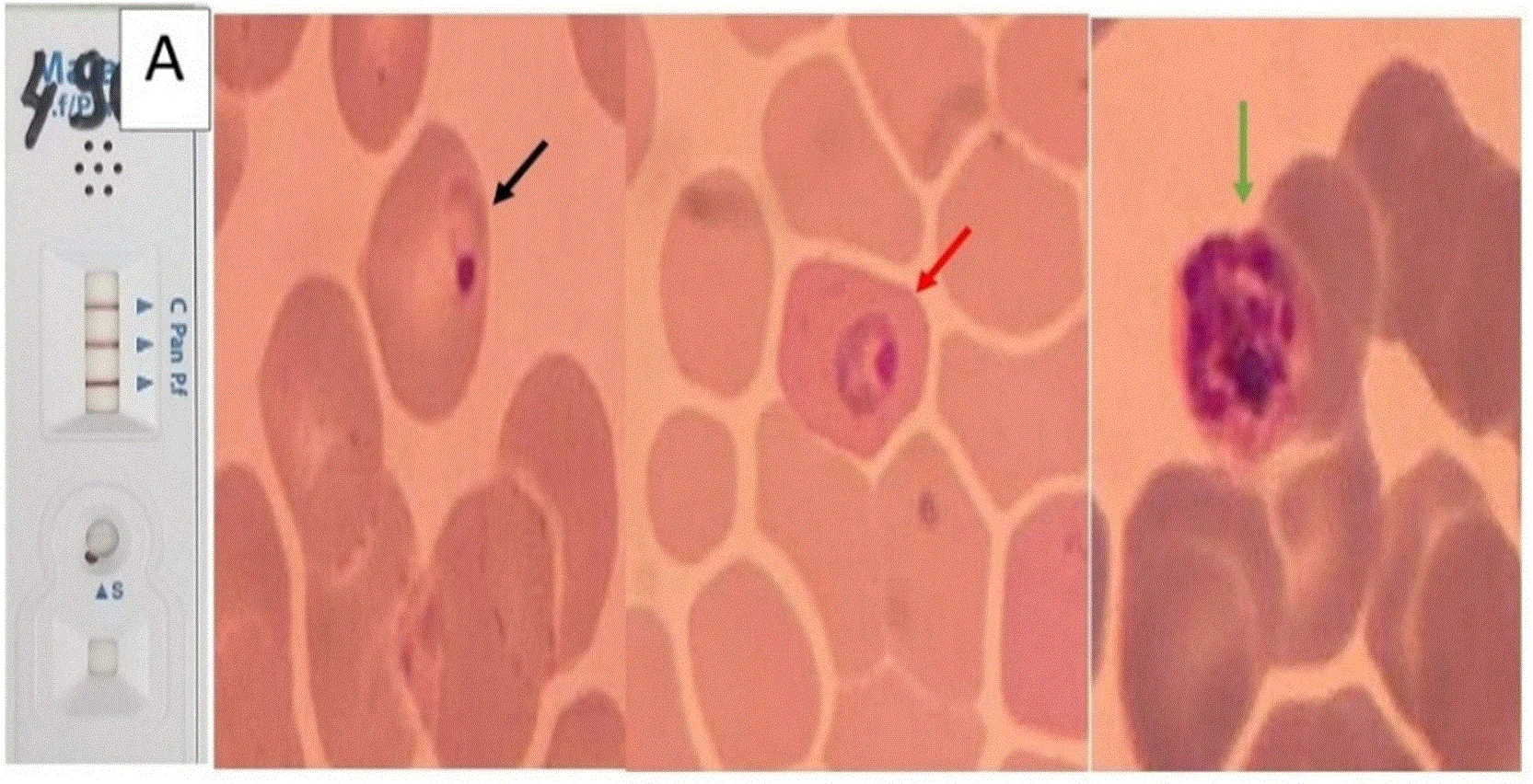
RDT and microscopic findings in case 4. A: PAN/Pf positive RDT; *P. falciparum* trophozoite (black arrow), *P. ovale* trophozoite (red arrow), and *P. ovale* schizont (green arrow) on thin blood smear.

Tables 1 and 2 summarized the main epidemiological, clinical, and biological features of the four patients. Figures 1, 2, 3, and 4 show the parasitological diagnostic results of patients 1 to 4, respectively, including patient-specific rapid diagnostic tests and blood smears.

**Table 1.** Epidemiological, clinical, and parasitological features of the four patients with mixed *Plasmodium* infections.

Patient	1	2	3	4
Date of diagnosis	25/01/2024	18/02/2024	20/02/2024	26/05/2025
Age/sex	33/M	52/M	31/M	39/F
Return from Central African Republic (days)	10	30	30	15
Clinical presentation	Fever 41°C, abdominal pain, headache, myalgia	Fever 42°C, chills, asthenia	Fever 40°C, headache, vomiting; 6 prior malaria episodes	Fever 38°C, headache, myalgia
RDT result	PAN (+)	PAN (+)	HRP2 (+), PAN (+)	HRP2 (+), PAN (+)
Blood smear findings	*P. falciparum + P. malariae* (trophozoites)	*P. ovale + P. malariae* (trophozoites, schizonts, gametocytes)	*P. falciparum* (trophozoites) + *P. ovale* (trophozoites, schizonts, gametocytes)	*P. falciparum* (trophozoites) + *P. ovale* (trophozoites, schizonts)
Parasitemia	1%	<1%	<1%	<1%
Treatment	Coartem ^®^	Coartem ^®^ + Primaquine	Coartem ^®^ + Primaquine	Coartem ^®^ + Primaquine
Outcome (D3-D7-D28 smears)	Negative	Negative	Negative	Rare degenerated trophozoites at D3 Negative at D7–D28

**Table 2.** Biological characteristics of the four patients with mixed *Plasmodium* infections.

Complete Blood count (CBC), differential white blood cells (WBCs) count and coagulation tests					
	Case 1	Case 2	Case 3	Case 4	Reference interval
Blood group	-	AB positif	O positif	AB negatif	-
Hemoglobin	14.6	11,7	15,2	14	12-16 g/dl
Hematocrit	42.9%	35.2		42	37-47%
Red blood cells	4.8	4.18		5.14	4.2-5.5 X 10 ^12^/L
MCV	89.3	84.2	83.3	81.7	80-94 fl
MCH	34.1	27.9	28	27.2	27-32 pg
White blood cell count X 10 ^9^/L	8.0	4.8	10.5	9.3	4-11 X 10 ^9^/L
Neutrophils X 10 ^9^/L	6.6	3.9	9.0	8.1	1.5-8 X 10 ^9^/L (40-75%)
Lymphocytes X 10 ^9^/L	0.9	0.4	1	0.6	1-4 X 10 ^9^/L (20-45%)
Monocytes X 10 ^9^/L	0.5	0.5	0.5	0.5	0.1-0.7 X 10 ^9^/L (3-9%)
Eosinocytes X 10 ^9^/L	00	00	00	00	0.05-0.5 X 10 ^9^/L (0-6%)
Basophils X 10 ^9^/L	00	00	00	0.1	0.025-0.1 X 10 ^9^/L (0-1%)
Platelets X 10 ^9^/L	86 X 10 ^9^/L	37 X 10 ^9^/L	87 X 10 ^9^/L	48 X 10 ^9^/L	140-450 X 10 ^9^/L
Coagulation tests					
Prothrombin time	-	61%		90%	70-100%
International Normalized ratio	-	1.37		1.05	2-3
Biochemistry, liver function tests and inflammatory blood markers tests					
C- reactive protein	144	142	109	123	<6 mg/L
Erythrocyte sedimentation rate					0-20 mm/hour
Total Protein	7.2	6.3	7.7	6.9	6.4-8.2 g/dL
Albumin					3.4-5.0 g/dL
Na/K	134/3.6	131/3.6	131/4.1	136/3.9	136-145 mEq/L/3.5-5.1 mEq/L
Urea	7.5	5.3	5.2	6.4	7-18 mg/dL
Creatinin	89	97	69	65	60-105 μmol/L
AST (Aspartate Aminotransferase)	17	136	19	45	15-37 U/L
ALT (Alanine Aminotransferase)	17	118	20	45	14-36 U/L
Alkaline phosphatase	65	208	96	58	46-116 U/L
Gamma-glutamyl transferase	34	516	42	38	5-55 U/L
Total bilirubin	29	37	21	10	<20 μmol/L
Direct bilirubin	3	19	-	-	<5,1 μmol/L

## Discussion

This study provides a detailed description of four MPI in military personnel returning from Central African Republic, with thorough documentation of clinical features, diagnostic tests, and follow-up parasitological clearance, which represents strength. Limitations include the small sample size, retrospective design, lack of molecular confirmation, and the exclusive focus on military personnel. Despite these constraints, the cases highlight that mixed infections can occur even under chemoprophylaxis, may present with low parasitemia, and require careful species identification.

MPI are underreported, but they are more common than previously thought, especially in regions where multiple species coexist. These infections may go undetected due to the dominance of one species or low parasitemia. Studies in African and Asian endemic areas report prevalence rates ranging from 2% to 30%, depending on local transmission intensity and the sensitivity of diagnostic tools. ^10^ ^,^ ^11^

In 2023, the WHO African Region accounted for 94% of global malaria cases and 95% of deaths, with Nigeria (25.9%) and the Democratic Republic of the Congo (12.6%) contributing the largest shares. The Central African Republic represented 0.6% of cases. In this context, mixed *Plasmodium* infections remain a notable concern due to the co-endemicity of *P. falciparum, P. ovale,* and *P. malariae.* ^12^

The prevalence of MPI in our study was 2.2%. The four cases reported included one involving *P. falciparum* and *P. malariae*, one involving *P. ovale* and *P. malariae*, and two involving *P. falciparum* and *P. ovale*.

In Tunisia, MPI remain poorly documented. Bouratbine A. et al. reported five cases of MPI among 240 *Plasmodium* infections (1980-1995), including *P. falciparum–P. ovale* (2 cases), *P. falciparum–P. malariae* (2 cases), and *P. falciparum–P. vivax* (1 case). ^13^ Belhadj et al. found six cases of MPI among 291 *Plasmodium* infections (1991-2006), with a similar distribution: *P. falciparum–P. ovale* (4 cases), *P. falciparum–P. malariae* (1 case), and *P. falciparum–P. vivax* (1 case). ^14^ More recently, Siala et al. reported two cases of MPI: *P. falciparum–P. ovale* and *P. falciparum–P. malariae.* ^8^

MPI can result from several factors. Multiple Anopheles species may transmit different *Plasmodium* species simultaneously in a single inoculation, or a single *Plasmodium* species may be introduced through successive bites by infected mosquitoes. *P. vivax* and *P. ovale* can persist as liver hypnozoites, causing relapses, while *P. falciparum* may recrudesce due to drug-resistant forms. Incomplete treatment of a prior infection can also lead to persistent or mixed parasitemia. These mechanisms highlight the complexity of diagnosing and managing mixed malaria infections in endemic areas. ^4^ ^–^ ^6^

Accurate identification of mixed infections is crucial for appropriate management. Peripheral blood smear microscopy remains the gold standard for species identification and quantification, allowing detection of multiple species. However, it may fail to detect low-density parasitemia or minor species. Rapid diagnostic tests (RDTs) are valuable for rapid screening, detecting pan- *Plasmodium* antigens and species-specific markers, but they have known limitations, as some minor infections may yield false-negative results. ^15^ ^,^ ^16^ Molecular techniques, such as PCR especially multiplex, nested approaches, or real-time PCR offer superior sensitivity and specificity, capable of identifying submicroscopic infections and clarifying complex species combinations. ^1^ ^,^ ^2^ ^,^ ^17^ ^,^ ^18^ In our study, molecular confirmation was not available; therefore, diagnosis relied on microscopy combined with RDT results. Metagenomic next-generation sequencing (mNGS) is a powerful tool for detecting mixed infections, useful in atypical or severe cases where routine tests fail, but its cost and complexity limit malaria use in endemic regions. ^19^ Incorporating multiple diagnostic approaches can improve detection and guide targeted therapy.

Treatment of mixed *Plasmodium* infections should aim to eliminate all infecting species and prevent relapses. In our cases, Coartem ^®^ was administered to all patients, complemented by a 15-day course of primaquine for *P. ovale* infections to target dormant liver stages. This combined approach resulted in rapid clinical recovery and confirmed parasitological clearance by day 28.

Effective management of mixed infections requires selecting antimalarial regimens active against all infecting species. Artemisinin-based combination therapies (ACT) are effective against both *P. falciparum* and non- *falciparum* species. Additional drugs, such as primaquine, are necessary to eliminate hepatic stages of *P. vivax* and *P. ovale.* ^20^ In our study, primaquine was administered without quantitative G6PD testing; patients were monitored using serial complete blood counts (CBC), and no hemolysis or adverse events were observed, supporting the safety of this approach in our patients.

Early intervention and adherence to recommended treatment protocols are critical, as untreated or inadequately treated mixed infections may lead to severe disease, prolonged illness, and contribute to the development of antimalarial resistance. ^20^ ^,^ ^21^ Our findings reinforce the importance of considering species-specific therapies and following WHO 2025 malaria treatment guidelines in co-endemic settings, particularly for relapsing species requiring radical cure. ^22^ Preventive strategies remain essential to reduce the incidence and impact of malaria, particularly for travelers and military personnel deployed in endemic areas. Effective measures include adherence to chemoprophylaxis, use of insecticide-treated bed nets, application of topical repellents, and prompt medical evaluation at the onset of fever or other malaria symptoms. Education and awareness of the risk of these infections are also vital. ^23^ Strengthening preventive programs and surveillance can help mitigate the burden of imported and co-endemic malaria cases.

## Conclusion

Mixed *Plasmodium* infections are often underdiagnosed and can present diagnostic and therapeutic challenges. In our case series, microscopy and rapid diagnostic tests allowed accurate species identification. Timely administration of appropriate ACT, complemented by primaquine for relapsing species, resulted in full recovery and parasitological clearance in all reported cases. Although not used in this study, molecular approaches are important for definitive species confirmation in mixed infections. These findings suggest that careful diagnostic evaluation and adherence to treatment guidelines are critical in managing mixed malaria infections, particularly in patients returning from endemic areas.

## Consent

Written informed consent for the publication of clinical details was obtained from all patients.

## Data Availability

All data underlying the results are included within the article, and the CARE Checklist is openly available in public repositories (Mtibaa L. CARE checklist – Mixed Plasmodium infection [Data set]. Zenodo; 2025. DOI: https://doi.org/10.5281/zenodo.17650886 under a CC0 1.0 license.)
