# Supplementary material for: Mixed Plasmodium Infections: Report of Four Cases from the Military Hospital of Tunis
Source: F1000Res. 2026 Sep 8;14:1389. Originally published 2025 Dec 10. [Version 3] doi: 10.12688/f1000research.173540.3 (PMC13583345; doi:10.12688/f1000research.173540.3)
Supplement: Peer review history

# Reviewer report: Reviewer response for version 3

**DOI:** 10.5256/f1000research.209513.r519191

**Published:** 2026-09-16

**Authors:** Felix Zoiku

**Affiliations:** 1 University of Ghana, Accra, Ghana

I do not have any further comment. I am satisfied with the responses from the authors.

Are enough details provided of any physical examination and diagnostic tests, treatment given and outcomes?

Yes

Is the background of the cases’ history and progression described in sufficient detail?

Yes

Is sufficient discussion included of the importance of the findings and their relevance to future understanding of disease processes, diagnosis or treatment?

Yes

Is the conclusion balanced and justified on the basis of the findings?

Yes

Reviewer Expertise:

Molecular Parasitology, Malaria Epidemiology, Drug discovery

I confirm that I have read this submission and believe that I have an appropriate level of expertise to confirm that it is of an acceptable scientific standard.

# Reviewer report: Reviewer response for version 3

**DOI:** 10.5256/f1000research.209513.r519645

**Published:** 2026-09-15

**Authors:** Loick P. Kojom Foko

**Affiliations:** 1 Animal Organisms, The University of Douala, Douala, Littoral, Cameroon

**Abstract**

- Malaria is caused by six species, not five. *Plasmodium ovale* has been split into two genetically distinct species, *P. ovale wallikeri* and *P. ovale curtisi*.
- This section needs improvement. The authors conducted the study in Tunisia. What mixed infections are a problem in Tunisia? Are single infections dominant in the country?
- Change ‘ *P. ovale*’ to ‘ *P. ovale* spp.’. The authors did not perform molecular analyses to distinguish between *P. ovale wallikeri* and *P. ovale curtisi*.
- Remove the commercial name ‘Coartem’
- The conclusion does not reflect the findings of this study.
- The keywords are incomplete. Terms such as ‘Tunis’ should be keywords.

**Introduction**

- The sentence ‘ **Malaria is a life-threatening vector-borne disease caused by five species of the genus *Plasmodium* (P.): *P. falciparum*, *P. vivax*, *P. malariae*, *P. ovale*, and *P. knowlesi*.** ’ should be revised as per my comments on the abstract.
- The sentence ‘ **Most imported cases originate from sub-Saharan Africa, particularly Côte d’Ivoire and the Democratic Republic of the Congo.**’ is misleading. The authors cited two small-scale studies to support this statement. I suggest rewording it.
- The following two sentences ‘ **Among Tunisian military personnel, few studies have specifically addressed MPI, and epidemiological data remain limited.9 A clear understanding of species distribution and epidemiological patterns is essential for accurate diagnosis, optimal treatment, and relapse prevention.**’ are not related. Do you think that you can understand the epidemiological patterns of mixed infections in a specific group (i.e., Military people)? The authors need to revise the rationale for this study; otherwise, I wonder why they did not work in hospitals receiving individuals returning from international travel.
- Again, the last sentence of the introduction is not connected with the real objective of this study. The authors discussed ‘diagnostic and therapeutic challenges’ of mixed infections, but nowhere in the introduction do they present a comprehensive state of the art of previous investigations into these challenges.

**Materials and Methods**

- I did not understand the need for the following sentence: ‘The denominator for prevalence calculation included all imported malaria cases diagnosed during the study period. ’.
- Did the ‘experienced microscopist ’ WHO-certified microscopists? If so, specify the WHO accreditation level. If not, how did you evaluate their experience to detect mixed infections?
- The authors need to provide a supporting reference for the following statement ‘ **An average of approximately 250 red blood cells per microscopic field was considered for this estimation**.’
- The authors need to present the detection performances of the RDT as per the latest WHO assessment round for malaria RDTs. The document is available online: https://www.who.int/publications/i/item/9789241514965.
- The authors need to provide a supporting reference for the following statement ‘ **in accordance with current WHO and Tunisian military health recommendations.**’
- The authors need to provide a supporting reference for the following statement ‘ **Those with P. ovale infection received an additional 15-day course of primaquine for radical cure, as recommended by the WHO.**’ Also, this sentence concludes that the authors expect to find *P. ovale*. *Plasmodium vivax* is also a relapsing species, and several studies in sub-Saharan Africa have reported growing evidence of its presence. The authors need to revise this sentence.

**Results**

- Change ‘ *P. ovale*’ to ‘ *P. ovale* spp.’ in the manuscript.
- The authors need to only present the results and avoid any interpretation. Please, remove the interpretations in this section.
- Table 2. Some digits display a comma instead of a dot. The authors are probably French-speaking.
- The authors have to improve the quality of the figures, as well as the design.

**Discussion**

- The authors need to provide references supporting this sentence ‘ **MPI are underreported, but they are more common than previously thought, especially in regions where multiple species coexist**.’
- The authors need to update some statistics with the World Malaria Report 2025.
- The sentence ‘ **The prevalence of MPI in our study was 2.2%.**’ is misleading. You mean the total number of MPI relative to the total number of Plasmodium-infected individuals, or to the total number of screened individuals?
- The authors listed previous studies which reported mixed infections in Tunisia. I would like to know what the prevalence range of mixed infections across these listed studies is.

Are enough details provided of any physical examination and diagnostic tests, treatment given and outcomes?

No

Is the background of the cases’ history and progression described in sufficient detail?

No

Is sufficient discussion included of the importance of the findings and their relevance to future understanding of disease processes, diagnosis or treatment?

No

Is the conclusion balanced and justified on the basis of the findings?

Partly

Reviewer Expertise:

Malaria, Tropical diseases, Epidemiology, Public Health, One Health, Biotechnology, Genetics.

I confirm that I have read this submission and believe that I have an appropriate level of expertise to confirm that it is of an acceptable scientific standard, however I have significant reservations, as outlined above.

# Reviewer report: Reviewer response for version 2

**DOI:** 10.5256/f1000research.197562.r507970

**Published:** 2026-08-27

**Authors:** Felix Zoiku

**Affiliations:** 1 University of Ghana, Accra, Ghana

**Mixed *Plasmodium* Infections: Report of Four Cases from the Military Hospital of Tunis**

**Overview**

 The manuscript is original, well structured, and clearly written, particularly with regard to the methodology, presentation of results, and interpretation of the data. Overall, the study is relevant and contributes useful information to the literature. However, a few minor issues require clarification before the manuscript can be considered for indexing.

 This is a retrospective descriptive study involving cases of mixed *Plasmodium* infections diagnosed at the Parasitology Laboratory of the Military Hospital of Tunis between 2022 and 2025. Diagnosis was established using May–Grünwald–Giemsa-stained thin blood smears, Giemsa-stained thick blood smears, and rapid diagnostic tests (RDTs). *Plasmodium* species were identified based on standard microscopic morphological criteria. The main objective was to describe the epidemiological, clinical, and biological characteristics of four cases of mixed *Plasmodium* infection.

 The authors report four cases of mixed *Plasmodium* infection among military personnel returning from deployment in the Central African Republic. The RDTs were positive for pan- *Plasmodium* pLDH in all four cases, while two patients were also positive for HRP2. Peripheral blood smear examination confirmed mixed infections, with parasitaemia generally low (<1% in three cases and 1% in one case).

 Regarding treatment, all patients received artemether–lumefantrine (Coartem®), with primaquine added for patients diagnosed with *P. ovale* infection. The patients were subsequently monitored through blood smear examinations on days 3, 7, and 28. These follow-up examinations confirmed parasitological clearance, with only rare degenerated trophozoites observed in one patient on day 3.

 The authors conclude that mixed *Plasmodium* infections may influence disease severity, treatment outcomes, and the potential emergence or detection of drug resistance. They appropriately emphasize the importance of accurate diagnosis and appropriate radical treatment, particularly for infections involving species such as *P. ovale*, to prevent relapse. Overall, the study represents a valuable addition to the existing literature, particularly because imported mixed malaria infections are relatively uncommon in Tunisia.

 One of the main limitations of the study is the very small sample size, with only four cases reported. However, this limitation is understandable and can be justified by the epidemiological context of Tunisia, which eliminated indigenous malaria transmission in 1979. Consequently, malaria cases encountered in Tunisia are predominantly imported, making the identification and careful characterization of imported cases particularly important. This is especially relevant for mixed infections involving different *Plasmodium* species, which may present diagnostic and therapeutic challenges.

 Another limitation is the exclusive focus on military personnel. Although this limits the generalizability of the findings to the wider population, the study population is nevertheless relevant. Military personnel may be deployed to circulate in endemic regions, including areas where multiple *Plasmodium* species circulate, and may subsequently return to countries where malaria is not endemic. Systematic screening, accurate diagnosis, and appropriate follow-up of returning personnel are therefore important for the timely detection and management of imported malaria cases and for preventing onward transmission.

 Overall, the manuscript addresses an important but relatively underreported clinical and epidemiological issue. Despite the limited number of cases, the study provides useful insights into mixed *Plasmodium* infections among returning military personnel and highlights the importance of maintaining diagnostic vigilance for imported malaria in countries that have eliminated local transmission. Subject to clarification of the minor issues identified, I consider the manuscript suitable for indexing.

**Title**

 The title is suitable for the study.

**Abstract**

 The abstract was well structured and contained a brief background, method, results, and conclusion.

**Introduction**

 The study objectives and outcome measures are clearly defined and are supported by relevant and appropriate literature.

**Method**

**The authors need to clarify the following:**

**Comment 1**

**1. Paragraph 2:** Although Giemsa-stained thick blood smears were prepared, they were not used for parasite density estimation. Instead, the authors reported parasitaemia as a percentage. In clinical malaria diagnosis, parasite density, expressed as the number of parasites per microliter of blood, is generally more informative for assessing the magnitude and potential severity of infection. The authors should therefore clarify the rationale for reporting percentage parasitaemia rather than parasite density. It would be helpful to explain whether there was a specific methodological or clinical reason for choosing percentage parasitaemia, particularly since parasite density could provide a more standardized quantitative measure for evaluating the severity of infection and facilitating comparison with established clinical thresholds.

**Comment 2**

**2.** **Regarding this paragraph:** The authors state that all four patients received malaria chemoprophylaxis with doxycycline (100 mg once daily), initiated 1–2 days before travel to an endemic area, continued throughout their deployment in the Central African Republic, and maintained for four weeks after returning, in accordance with current WHO and Tunisian military health recommendations. The authors should clarify the source of this information. Was the chemoprophylaxis history documented in the patients’ hospital medical records, or was this information obtained through the structured medical interviews conducted as part of the study? If it was obtained through patient interviews, the authors should indicate this explicitly in the Methods section.

**Results**

**Comment 3**

 3. The authors should clarify whether the *Plasmodium* images presented in Figures 1, 2, 3, and 4 were obtained directly from the patients’ blood samples examined as part of this study. If the images were sourced from another publication, database, or external reference, the appropriate source should be clearly cited in the figure legends and/or the main text.

**Discussion**

 The discussion is well written and includes adequate literature.

**Conclusion**

 The conclusion was appropriate.

**References**

 The references were well put together with appropriate reference style

**Recommendation**

 This work is recommended for indexing under the condition that the above comments are addressed properly.    

Are enough details provided of any physical examination and diagnostic tests, treatment given and outcomes?

Yes

Is the background of the cases’ history and progression described in sufficient detail?

Yes

Is sufficient discussion included of the importance of the findings and their relevance to future understanding of disease processes, diagnosis or treatment?

Yes

Is the conclusion balanced and justified on the basis of the findings?

Yes

Reviewer Expertise:

Molecular Parasitology, Malaria Epidemiology, Drug discovery

I confirm that I have read this submission and believe that I have an appropriate level of expertise to confirm that it is of an acceptable scientific standard, however I have significant reservations, as outlined above.

# Response

**Published:** 2026-08-27

**Authors:** Latifa Mtibaa

**Affiliations:** Laboratory of Parasitology, Hopital Militaire Principal d'Instruction de Tunis, Tunis, Tunis, Tunisia

**Response to reviewer 2**

 We sincerely thank Dr. Felix Zoiku for his careful review of our manuscript and for his constructive and valuable comments. We have carefully considered each of the points raised and have revised the manuscript accordingly. We believe that these revisions have improved the clarity, accuracy, and overall quality of the manuscript. Our detailed responses to each comment are provided below, with the corresponding changes highlighted in the revised manuscript.

**Comment 1**

 1.Paragraph 2: Although Giemsa-stained thick blood smears were prepared, they were not used for parasite density estimation. Instead, the authors reported parasitaemia as a percentage. In clinical malaria diagnosis, parasite density, expressed as the number of parasites per microliter of blood, is generally more informative for assessing the magnitude and potential severity of infection. The authors should therefore clarify the rationale for reporting percentage parasitaemia rather than parasite density. It would be helpful to explain whether there was a specific methodological or clinical reason for choosing percentage parasitaemia, particularly since parasite density could provide a more standardized quantitative measure for evaluating the severity of infection and facilitating comparison with established clinical thresholds.

**Response**

 We thank the reviewer for this valuable comment.

 For all four patients, both thick and thin blood smears were systematically prepared and examined. The thick blood smear was used primarily for parasite detection, while the thin blood smear was used for morphological species identification and estimation of parasitaemia.

 In our study, parasitaemia was expressed as the percentage of parasitized red blood cells. It was quantitatively estimated on the thin blood smear using the following formula:

**Parasitaemia (%) = [number of parasitized red blood cells (RBCs) / (number of RBCs per field × number of fields examined)] × 100.**

**An average of approximately 250 red blood cells per microscopic field was considered for this estimation.**

 We acknowledge that parasite density expressed as the number of parasites per microliter of blood provides a standardized quantitative measure and is commonly used for assessing malaria parasite burden and disease severity. However, parasite density in parasites/µL was not directly determined in this retrospective case series. We therefore considered it more appropriate to report the parasitaemia values that were actually measured on the thin blood smears rather than retrospectively deriving parasite densities from incomplete or unavailable quantitative data.

 We have clarified this point in the Methods section by describing the respective roles of the thick and thin blood smears and the formula used for parasitaemia estimation.

**Comment 2**

 2. Regarding this paragraph: The authors state that all four patients received malaria chemoprophylaxis with doxycycline (100 mg once daily), initiated 1–2 days before travel to an endemic area, continued throughout their deployment in the Central African Republic, and maintained for four weeks after returning, in accordance with current WHO and Tunisian military health recommendations. The authors should clarify the source of this information. Was the chemoprophylaxis history documented in the patients’ hospital medical records, or was this information obtained through the structured medical interviews conducted as part of the study? If it was obtained through patient interviews, the authors should indicate this explicitly in the Methods section.

**Response**

 We thank the reviewer for this helpful comment. Information regarding malaria chemoprophylaxis, including the regimen, timing, duration, and adherence, was obtained through structured medical interviews conducted upon return. Compliance was self-reported and could not be objectively verified. We have clarified this point in the Methods section.

**Comment 3**

 The authors should clarify whether the *Plasmodium* images presented in Figures 1, 2, 3, and 4 were obtained directly from the patients’ blood samples examined as part of this study. If the images were sourced from another publication, database, or external reference, the appropriate source should be clearly cited.

**Response**

 Thank you for this helpful comment. We confirm that all microscopic images presented in Figures 1–4 are original images obtained directly from the thin blood smears of the four patients included in this study and were captured during the microscopic examination performed in our laboratory for species identification. We have revised the figure legends to provide clearer descriptions of the rapid diagnostic tests and microscopic findings for each case. We have also clarified in the Results section that Figures 1,2,3, and 4 correspond to the patient-specific parasitological diagnostic results of the four cases 1,2,3, and 4, respectively, including the respective rapid diagnostic tests and blood smears.

# Reviewer report: Reviewer response for version 1

**DOI:** 10.5256/f1000research.191370.r448073

**Published:** 2026-02-04

**Authors:** Manju Rahi

**Affiliations:** 1 Indian Council of Medical Research—Vector Control Research Centre (ICMR-VCRC), Puducherry, Puducherry, India

This is an interesting article by Latifa et al on mixed *Plasmodium* infections in Tunisia. The four case reports of mixed infections are described in detail. Clinico-epidemiological characterisation of mixed infections, which are often not diagnosed correctly, is important, and such results need to be catalogued. Therefore, this study is an important addition to the literature. I have some minor concerns which should be addressed:

1. Four different cases of imported mixed *Plasmodium* infections were reported among 177 patients. Why were these infections associated with low parasitaemia despite symptoms? Which *Plasmodium* species were dominant in each case (as each patient was infected with two species)? What was the proportion of species-wise mono-infections among the remaining patients?
2. Six prior episodes in patient 3 were described as relapses due to *P. ovale*. Please describe the treatment history of this patient.
3. What chemoprophylaxis was used by the patients while travelling to endemic areas? Please describe this in detail.
4. Figure 4 has a typographical error: please change “Black arow” to “Black arrow.”
5. Please standardise drug formatting and use “Coartem®” consistently instead of “Coartem.”
6. Please provide details on the diagnostic methods used, including the criteria for morphological identification and the brands of rapid diagnostic tests (RDTs) used for both Pan and HRP2 detection.
7. Please use consistent nomenclature throughout the manuscript, for example “ *Plasmodium ovale*” instead of “ *P. ovale*.”
8. Please provide a statement on Institutional Review Board (IRB) approval or waiver for this retrospective study.
9. Please correct the prevalence estimate: 4/175 equals 2.3% (not 2.2%), and clearly state that the denominator refers to imported malaria cases.
10. Please complete the sentence correctly by replacing “with sex ratio 3. They presented…” with “with a sex ratio of 3M:1F. They presented…”
11. Although no adverse events were reported, high-dose primaquine requires monitoring. Please add details on safety monitoring and include G6PD test results, if performed.
12. Please update the references according to WHO 2025 guidelines.
13. The introduction omits key aspects of malaria epidemiology in Tunisia (imported cases, previous reports of mixed *Plasmodium* infections) and the Central African context of species distribution and transmission, which weakens the justification for this case series. The problem statement could be strengthened, for example: “Despite chemoprophylaxis, Tunisian military forces face persistent risk of mixed *Plasmodium* infections in co-endemic Central African regions, with underreported local challenges.”
14. The manuscript states that all patients adhered to chemoprophylaxis but omits critical details such as the specific regimens used, dosing and timing, assessment of adherence, and individual-level preventive measures (e.g., bed nets, repellents). This limits the assessment of breakthrough mixed *Plasmodium* infections despite prophylaxis. The authors should consider adding these details to Table 1.
15. The conclusion makes generalisations beyond the scope of this small case series of four patients. As a case series, the authors should avoid broad claims regarding diagnostic and prognostic challenges of mixed *Plasmodium* infections, particularly as no comparison with single-species infections was performed and all cases had favourable outcomes.
16. Please revise the conclusion accordingly.
17. The authors emphasise the importance of multiple diagnostic approaches (microscopy, RDTs, and molecular methods when available), citing literature on PCR detection of submicroscopic co-infections missed by routine diagnostics. However, the present study does not demonstrate such a multimodal approach, as it relies solely on microscopy and RDTs without molecular confirmation, even in cases of low parasitaemia (<1%), which may have led to under-detection of minor species.

Are enough details provided of any physical examination and diagnostic tests, treatment given and outcomes?

Partly

Is the background of the cases’ history and progression described in sufficient detail?

Yes

Is sufficient discussion included of the importance of the findings and their relevance to future understanding of disease processes, diagnosis or treatment?

Partly

Is the conclusion balanced and justified on the basis of the findings?

Partly

Reviewer Expertise:

Vector-borne diseases specialised in Malaria

I confirm that I have read this submission and believe that I have an appropriate level of expertise to confirm that it is of an acceptable scientific standard, however I have significant reservations, as outlined above.

# Response

**Published:** 2026-03-09

**Authors:** Latifa Mtibaa

**Affiliations:** Laboratory of Parasitology, Hopital Militaire Principal d'Instruction de Tunis, Tunis, Tunis, Tunisia

**Response to reviewer 1**

 We sincerely thank Dr. Manju Rahi for her careful review of our manuscript and for her constructive and insightful comments. We have revised the manuscript accordingly and respond point-by-point below.

**Comment 1**

*Why were these infections associated with low parasitaemia despite symptoms? Which species were dominant? What was the proportion of mono-infections among the remaining patients?*

**Response:**

 We agree this point required clarification. Low parasitaemia despite symptoms may be explained by partial immunity related to repeated exposure in endemic areas, prior malaria episodes (notably in patient 3), and chemoprophylaxis use, which may attenuate parasite multiplication without preventing infection.

 In our series, *Plasmodium falciparum* was morphologically dominant in cases 1, 3, and 4, whereas *Plasmodium ovale* predominated in case 2.

 Among the remaining 171 imported malaria cases, species distribution was as follows:

- *Plasmodium falciparum*: 40%
- *Plasmodium ovale*: 57%
- *Plasmodium malariae*: 3%

These data have now been added to the Results section.

**Comment 2**

*Treatment history of patient 3 (six prior relapses).*

**Response:**

 We have added detailed treatment history for patient 3. Previous episodes were treated with artemisinin-based combination therapy without primaquine in earlier episodes, which may explain relapses related to dormant liver hypnozoites. This clarification has been incorporated into the Results section.

**Comment 3**

*Chemoprophylaxis details.*

**Response:**

 All four patients received malaria chemoprophylaxis with doxycycline (100 mg once daily), initiated 1–2 days before travel to endemic areas, continued throughout deployment in Central Africa, and maintained for four weeks after return, in accordance with current WHO and Tunisian military health recommendations. Adherence was assessed through structured medical interviews upon return; compliance was self-reported and could not be objectively verified.

 In addition to chemoprophylaxis, preventive measures included the use of insecticide-treated bed nets, topical mosquito repellents (DEET-based), and protective clothing.

 These details have been added in the Methods section.

**Comment 4**

 Typographical error corrected (“Black arrow”).

**Comment 5**

 Standardisation of drug formatting.

**Response:**

 We have standardised the use of “Coartem ^®^” throughout the manuscript.

**Comment 6**

 Diagnostic methods details and RDT brand.

**Response:**

 We have expanded the Methods section to include:

- Morphological criteria for species identification (size and shape of trophozoites, Schüffner’s stippling, band forms, schizont morphology, gametocyte characteristics).
- Rapid diagnostic tests were performed using the **STANDARD™ Q Malaria P.f/Pan Ag (SD Biosensor®)** **,** which detects histidine-rich protein 2 (HRP2) specific for *Plasmodium falciparum* and pan- *Plasmodium* lactate dehydrogenase (pLDH). Tests were conducted according to the manufacturer’s instructions.

**Comment 7**

 IRB approval.

**Response:**

 An ethics statement has now been added to the Methods section. As this was a retrospective study using fully anonymised patient data, formal Institutional Review Board approval was not required according to institutional regulations. The study was conducted in accordance with the principles of the Declaration of Helsinki.

**Comment 8**

 Prevalence correction.

**Response:**

 We thank the reviewer for noting this. The prevalence has been corrected to 2.3% (4/175), and we clearly state that the denominator refers to imported malaria cases.

**Comment 9**

 Sex ratio correction.

**Response:**

 The sentence has been revised to:

 “Patients were aged 31–52 years (mean age 38.8 years) with a sex ratio of 3 M: 1 F.”

**Comment 10**

 Primaquine safety and G6PD testing.

**Response:**

 We have clarified that patients receiving primaquine were monitored by serial complete blood counts (CBC) to detect any signs of hemolysis. No quantitative G6PD testing was performed. During treatment, no hemolysis or significant changes in hemoglobin levels were observed. Safety monitoring details have been added to the Methods and Results sections.

**Comment 11**

 WHO 2025 update.

**Response:**

 We have updated the manuscript to reflect the latest WHO 2025 malaria treatment guidelines, particularly regarding the use of ACTs and radical cure recommendations for relapsing species

**Comment 12**

 Strengthening epidemiological background.

**Response:**

 Thank you for this comment. We have strengthened the epidemiological background in both the Introduction and Discussion sections. Updated WHO 2023 data were included, highlighting that the African Region accounts for 94% of global malaria cases and 95% of malaria-related deaths. We also provided specific context for the Central African Republic to situate the study population. Additionally, we reinforced the relevance of mixed *Plasmodium* infections by discussing their prevalence, diagnostic challenges, and potential clinical implications in high-burden regions.

**Comment 13**

 Preventive measures and adherence details.

**Response:**

 Additional details regarding chemoprophylaxis regimens, adherence assessment, and personal preventive measures have been added in the Methods section.

**Comment 14–15**

 Overgeneralisation in conclusion.

**Response:**

 We have revised the conclusion to avoid broad generalisations. The revised conclusion now emphasises that our findings are limited to a small case series and should be interpreted cautiously, without extrapolation beyond the presented cases.

**Comment 16**

 Absence of molecular confirmation.

**Response:**

 Thank you for this comment. We acknowledge that molecular methods could improve the confirmation of mixed *Plasmodium* infections. However, these techniques were not available in our laboratory during the study period. This limitation has now been clearly acknowledged in the Discussion section.

 We thank the reviewer again for her valuable comments, which have significantly strengthened our manuscript.
